# A Phase IB Trial of Selinexor in Combination With Immune Checkpoint Blockade in Patients With Advanced Renal Cell Carcinoma

**DOI:** 10.1002/cam4.70280

**Published:** 2025-02-13

**Authors:** Omar Alhalabi, Mohamed A. Gouda, Denái R. Milton, Hassan Ahmed Momin, Bulent Yilmaz, Bettzy Stephen, Chinenye Lynette Ejezie, Justin Tyler Moyers, Serdar A. Gurses, Jeffrey How, Siqing Fu, Jordi Rodon, David S. Hong, Sarina A. Piha‐Paul, Vivek Subbiah, Ecaterina Elena Dumbrava, Daniel D. Karp, Filip Janku, Funda Meric‐Bernstam, Nizar M. Tannir, Aung Naing

**Affiliations:** ^1^ Department of Genitourinary Medical Oncology, Division of Cancer Medicine The University of Texas MD Anderson Cancer Center Houston Texas USA; ^2^ Department of Investigational Cancer Therapeutics, Division of Cancer Medicine The University of Texas MD Anderson Cancer Center Houston Texas USA; ^3^ Department of Biostatistics The University of Texas MD Anderson Cancer Center Houston Texas USA; ^4^ The Angeles Clinic and Research Institute, a Cedars‐Sinai Affiliate Los Angeles California USA

**Keywords:** immunotherapy, renal cell carcinoma, selinexor

## Abstract

**Background:**

Selinexor (SEL) is a nuclear exportin 1 inhibitor that blocks the transport of nuclear proteins, including tumor suppressors, to the cytoplasm. Preclinical data suggest that the combination of SEL with checkpoint blockade may result in improved response to immunotherapy.

**Methods:**

NCT02419495 was a multiarm phase IB study of SEL in combination with other standard regimens in patients with advanced malignancies. Arm M utilized twice weekly oral SEL and intravenous nivolumab (NIVO). Arm N utilized weekly oral SEL with NIVO plus ipilimumab (IPI). The primary objective of this study was to evaluate the safety of SEL + NIVO and SEL + NIVO+IPI. Secondary objectives included determining the objective response rate (ORR) and progression‐free survival (PFS).

**Results:**

Twenty‐nine patients were enrolled in the study, of which 26 (90%) had clear cell RCC (ccRCC). Most patients (72%, *n* = 21) had prior systemic therapies. All patients (100%) developed at least one treatment‐emergent adverse event, and 93% had a treatment‐related adverse event (TRAE). Grade ≥ 3 TRAE occurred in 31% of patients, including 10% with hyponatremia, 7% with neutropenia, and 7% with thromboembolic events. At a median follow‐up of 12.4 months, the ORR in 27 patients evaluable for response was 19% (*n* = 5). An additional 17 patients (63%) had stable disease (SD) as the best response. The median PFS for the overall cohort was 14.5 months (95% CI 5.2–17.4 months; SEL + NIVO+IPI: 12.2 months, SEL + NIVO: 14.5 months). The median overall survival was 27.8 months (95% CI 15.3–32.5; SEL + NIVO+IPI: unreached, SEL + NIVO: 21.3 months).

**Conclusions:**

SEL in combination with NIVO or NIVO+IPI had a potentially favorable safety profile and showed modest clinical activity in patients with advanced renal cell carcinoma.

**Trial Registration:** This clinical trial was registered on clinicaltrials.gov (NCT02419495)

## Graphical abstract

Treatment with selinexor in combination with nivolumab or nivolumab plus ipilimumab is well‐tolerated. In patients with advanced renal cell carcinoma, the combination showed modest clinical activity compared to historic immunotherapy alone.

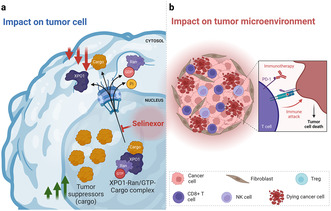

## 1 Introduction

The translocation of proteins, including tumor suppressor proteins and growth‐regulating oncoproteins, from the nucleus to the cytoplasm is an essential process for normal cell functioning and development. Exportins, a group of proteins responsible for moving different molecules across the nuclear membrane, therefore play a key role in cellular homeostasis [1, 2]. Among these, exportin 1 (XPO1) is one of the most extensively studied, facilitating the transfer of more than 200 distinct proteins [3, 4, 5, 6, 7]. XPO1 overexpression, can hence be associated with tumorigenesis due to the upregulated transfer of tumor suppressor proteins to the cytoplasm [8]. This has been reported in multiple studies to drive poor prognosis in both solid and hematological malignancies [1, 9, 10, 11, 12].

**FIGURE 1:**
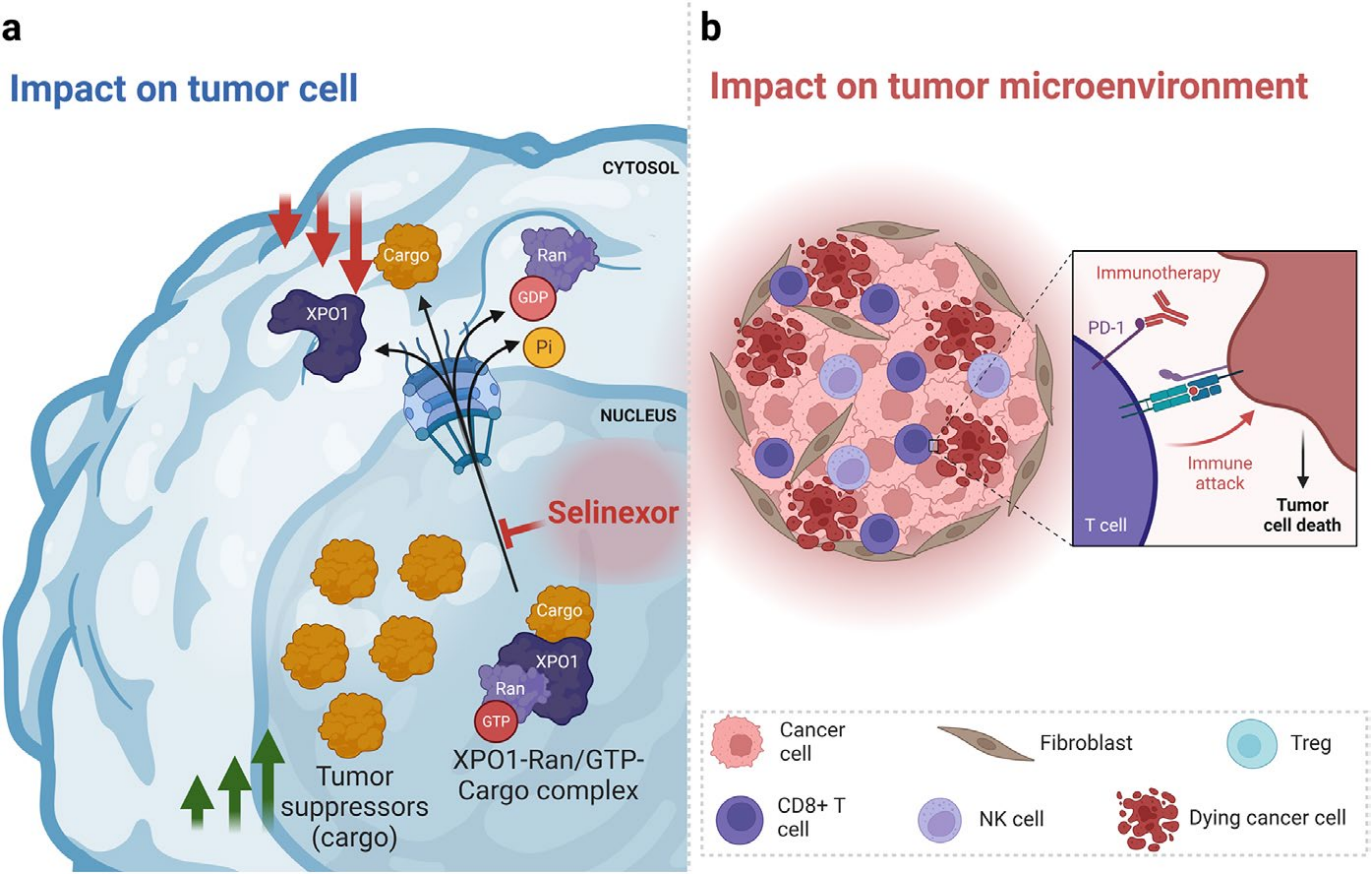
Rationale for selinexor in combination with immunotherapy. (a) Selinexor leads to a buildup of tumor suppressors (e.g., p53, RB1, p21) in the nucleus of malignant cells and reduce levels of oncogene products which drive cell growth. (b) The combination with immunotherapy may lead to changes in the tumor microenvironment that enhances the response to immunotherapy. In a syngeneic mouse model of RCC, the combination of selinexor with checkpoint blockade antibody resulted in reorganization of the tumor immune landscape, with increases in the total number of T cells, effector and activated T cells, and NKT cells and reductions in myeloid cells and Tregs. Figure was made using tools from biorender.com.

Selective inhibitors of nuclear export (XPO1 inhibitors) block the transport of proteins leading to the accumulation and “re‐activation” of tumor suppressor proteins within the nucleus. Consequently, the translation of proto‐oncogenes is prevented, and the activity of the cell cycle checkpoint is regained; which eventually results in tumor cell apoptosis. Selinexor (KPT‐330) is a potent oral XPO1 inhibitor that inhibits XPO1 by forming a covalent bond with the cysteine 528 residue of the XPO1 cargo‐binding pocket [13, 14, 15, 16]. Preclinically, Selinexor (SEL) led to tumor growth inhibition by blocking the XPO1‐mediated nuclear transport. In particular, renal cell carcinoma (RCC) cell lines (ACHN and 786‐O) showed in vitro activity of SEL [17]. Furthermore, a xenograft mouse model using ACHN cell line showed inhibition of tumor growth when using SEL via increased nuclear localization of p21 [17]. As a single agent, preclinical and early clinical trials of SEL have demonstrated safety and clinical activity [13, 14, 15, 16, 18, 19, 20]. Additionally, in immune‐competent syngeneic mouse models, SEL combined with immune checkpoint blockade (ICB) resulted in a reduction of tumor burden via increased effector‐activated T cells, and NKT cells and reductions in myeloid cells and T‐regs (Figure 1) [21, 22]. This is probably driven by suppressing the nuclear export of STAT3, NFATc1, and other immunomodulatory molecules involved in the regulation of immune checkpoints; which in turn leads to superior efficacy of ICB [23, 24]. Notably, multiple therapeutic regimens including ICB are currently approved in patients with RCC given the promising results in clinical trials. However, primary or secondary resistance to ICB remains a clinical challenge necessitating the exploration of novel combinations with possible clinical benefit [25]. Taken together, we hypothesized that SEL combined with ICB is well tolerated and active against RCC. Therefore, we designed a phase IB clinical trial (NCT02419495) to investigate the safety of combined SEL with ICB in advanced solid tumors. In this article, we report the results from the RCC cohort.

## 2 Patients and Methods

### 2.1 Study Design and Treatment Plan

NCT02419495 was an open‐label, multiarm, investigator‐initiated, single‐center phase IB study of SEL with multiple standard chemotherapy or ICB regimens in patients with advanced malignancies. The trial used a 3 + 3 design for dose escalation and a “basket type” expansion. The primary objective was to determine the incidence of adverse events including dose‐limiting toxicities (DLTs), the maximum tolerated dose (MTD), and the recommended Phase 2 dose (RP2D) of SEL + NIVO and SEL + NIVO+IPI. Secondary objectives included the evaluation of preliminary antitumor activity. The MTD was defined as the highest dose level at which ≤ 33% of patients had DLTs during the first treatment cycle. After the MTD was determined, we performed dose expansion to include additional patients at the MTD level. The study protocol including subsequent modifications was approved by the Institutional Review Board at MD Anderson Cancer Center and. All study‐related procedures were conducted in accordance with the Declaration of Helsinki, Good Clinical Practice, and all local and federal regulatory guidelines. All subjects provided written informed consent prior to study enrollment.

Arm M utilized twice weekly oral SEL and intravenous nivolumab (NIVO). SEL was given at a dose of 40 mg twice a week (dose level 1 M, *N* = 8) or 60 mg twice a week (dose level 2 M, *N* = 11) with intravenous NIVO at a dose of 240 mg every 2 weeks or 480 mg every 4 weeks. Arm N utilized weekly oral SEL with NIVO plus ipilimumab (IPI). SEL was given at a dose of 60 mg (dose level 1 N, *N* = 4) or 80 mg (dose level 2 N, *N* = 6) weekly with NIVO 3 mg/kg × 4 cycles then 480 mg Q4W plus IPI 1 mg/kg Q3W for 4 cycles only.

### 2.2 Patient Population

All patients had a histologically confirmed advanced or metastatic solid cancer. Prior systemic therapy was not mandated, and there was no limit to the number of previous lines of treatment. All included patients had at least one measurable target lesion according to RECIST v1.1. Dose expansion was performed in patients with advanced RCC. Included patients were required to have adequate bone marrow, liver, and renal functions. Patients with primary central nervous system (CNS) tumor or active CNS involvement, residual toxicity from prior therapy that has not resolved to ≤ Grade 1 (excluding alopecia), prior treatment with an agent targeting the exportin, or medical conditions that could have affected safety or study outcomes were excluded from this study.

### 2.3 Assessments

Throughout the study and for 30 days following the conclusion of the treatment period, the occurrence of toxicities was monitored in all included patients. Treatment‐emergent adverse events (TEAE) and treatment‐related adverse events (TRAE) were graded using the Common Terminology Criteria for Adverse Events version 4.03 (CTCAE v4.03) A safety monitoring committee consistently reviewed all safety data and made consensus decisions during dose escalation. All patients had radiographic disease assessment with computerized tomography (CT) or magnetic resonance imaging (MRI) scans using RECIST v1.1 criteria every three cycles [26].

### 2.4 Statistical Methods

Descriptive statistics were used to summarize TEAEs, TRAEs, and antitumor activity. Objective response rate (ORR) was defined as the proportion of patients with complete or partial responses. Clinical benefit rate (CBR) was defined as the proportion of patients with partial response or stable disease for longer than 6 months. PFS was calculated from the time of treatment initiation to the time of disease progression or death, whichever occurred first, or off study. Patients who were alive and did not progress were censored at the last follow‐up or contact. Overall survival (OS) was calculated from the time of treatment initiation to the time of death. Alive patients were censored at the last follow‐up or contact. PFS and OS were estimated using Kaplan–Meier analysis. All analyses were performed in SAS 9.4 (Cary, NC).

## 3 Results

### 3.1 Patient Characteristics

**FIGURE 2:**
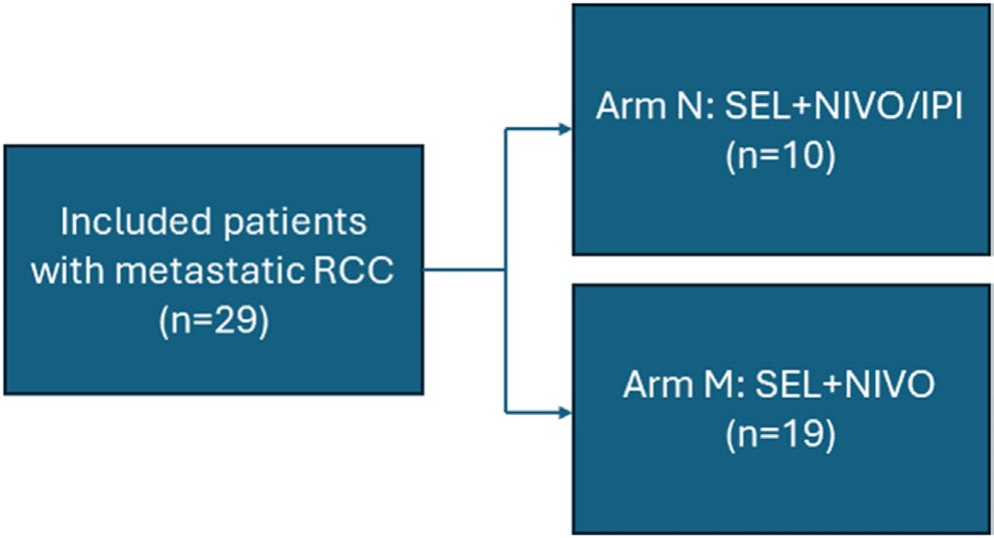
Included patients and different treatment groups. A total of 29 patients with metastatic RCC received treatment with selinexor in combination with nivolumab and ipilimumab (arm N; *n* = 10) or nivolumab monotherapy (arm M; *n* = 19).

Between January 2017 and March 2021, 29 patients with RCC were enrolled in our study (Table 1), including 3 (10%) patients with nonclear cell RCC (high‐grade sarcomatoid malignant neoplasm, poorly differentiated carcinoma with papillary features, and renal medullary carcinoma). Ten patients were treated in arm N with SEL + NIVO/IPI and 19 patients were treated in arm M with SEL + NIVO (Figure 2). Twenty‐five (86%) patients were males and 7 (24%) were of non‐White race. Median age at treatment initiation (C1D1) was 64 (IQR 56–69) years. Twenty‐one patients (72%) had prior systemic therapy, including 9 (31%) with a prior anti‐PD‐1/PD‐L1. Sixteen (55%) patients had two or more prior lines of therapy. During the dose escalation phase, four patients were treated at dose level 1 N, three patients were treated at dose level 1 M, six patients were treated at dose level 2 N, and two patients were treated at dose level 2 M. Fourteen patients were treated in the expansion phase of arm M, five of which were treated at dose level 1 M and nine were treated at dose level 2 M.

**TABLE 1:** Summary of patient and clinical characteristics.

Characteristics	All patients (*n* = 29)	Treatment arm	
		Selinexor + nivolumab + ipilimumab (*n* = 10)	Selinexor + nivolumab (*n* = 19)
Age at C1D1 (years)			
Median	64.3	62.0	67.7
Range	30.7–79.0	41.7–73.8	30.7–79.0
Gender, *n* (%)			
Male	25 (86)	7 (70)	18 (95)
Female	4 (14)	3 (30)	1 (5)
Race/ethnicity, *n* (%)			
White	22 (76)	9 (90)	13 (68)
Hispanic/Latino	4 (14)	0	4 (21)
Black	2 (7)	0	2 (11)
Unknown	1 (3)	1 (10)	0
ECOG performance status, *n* (%)			
0	0	0	0
1	29 (100)	10 (100)	19 (100)
RCC histopathology, *n* (%)			
Clear cell type	26 (90)	8 (80)	18 (95)
Renal medullary carcinoma	1 (3)	0	1 (5)
High‐grade sarcomatoid malignant neoplasm	1 (3)	1 (10)	0
Metastatic poorly differentiated carcinoma w/ papillary features	1 (3)	1 (10)	0
Prior lines of systemic therapies, *n* (%)			
0–1	13 (45)	8 (80)	5 (26)
2–3	11 (38)	2 (20)	9 (47)
4–5	3 (10)	0	3 (16)
> 5	2 (7)	0	2 (11)
Prior anti‐PD‐1/PD‐L1 therapy, *n* (%)			
No	20 (69)	8 (80)	12 (63)
Yes	9 (31)	2 (20)	7 (37)
Liver metastasis at baseline, *n* (%)			
No	23 (79)	10 (100)	13 (68)
Yes	6 (21)	0	6 (32)
Peritoneal metastasis at baseline, *n* (%)			
No	27 (93)	9 (90)	18 (95)
Yes	2 (7)	1 (10)	1 (5)
Lung metastasis at baseline, *n* (%)			
No	9 (31)	3 (30)	6 (32)
Yes	20 (69)	7 (70)	13 (68)
Lymph node metastasis at baseline, *n* (%)			
No	11 (38)	3 (30)	8 (42)
Yes	18 (62)	7 (70)	11 (58)
Bone metastasis at baseline, *n* (%)			
No	21 (72)	7 (70)	14 (74)
Yes	8 (28)	3 (30)	5 (26)

### 3.2 Safety and Tolerability

Dose level 1 on arm M was considered the RP2D due to long‐term tolerability. All 29 patients had at least one TEAE (Table 2). One patient died (Grade 5 TEAE) on study secondary to an unrelated adverse event of a blood clot. The most prevalent TEAEs of any grade (Table 3) were nausea (79%), increased creatinine (55%), anemia (52%), hyponatremia (52%), decreased platelets (48%), fatigue (41%), vomiting (38%), decreased white blood cell count (38%), hyperkalemia (34%), anorexia (31%), dyspnea (31%), and aspartate aminotransferase increased (24%). The most prevalent TRAEs of any grade (Table 3) were nausea (72%), decreased platelets (34%), fatigue (34%), vomiting (34%), hyponatremia (31%), decreased white blood cell count (28%), anemia (24%), and anorexia (24%). The most frequent Grade 3/4 TRAE were hyponatremia (10%), decreased neutrophil count (7%), and thromboembolic events (7%). Eight (28%) patients reported having serious adverse events (SAE), one of which (G3 transaminitis in a patient who received SEL + NIVO) was deemed related to treatment intervention (TRSAE) and led to discontinuation of therapy.

**TABLE 2:** Summary of treatment‐emergent adverse events.

Measure, *n* (%)	All patients (*n* = 29)	Treatment arm	
		Selinexor + nivolumab + ipilimumab (*n* = 10)	Selinexor + nivolumab (*n* = 19)
≥ 1 TEAE	29 (100)	10 (100)	19 (100)
≥ 1 TRAE	27 (93)	9 (90)	18 (95)
Grade 3/5 TEAE	18 (62)	6 (60)	12 (63)
Grade 3/5 TRAE	9 (31)	1 (10)	8 (42)
SAE	8 (28)	3 (30)	5 (26)
≥ 1 TRSAE	1 (3)	0 (0)	1 (5)
Discontinued due to ≥ 1 TEAE	0	0	0

Abbreviations: SAE, serious adverse events; TEAE, treatment‐emergent adverse events; TRAE, treatment‐related adverse events; TRSAE, treatment‐related serious adverse events.

**TABLE 3:** Summary of treatment‐emergent and treatment‐related adverse events.

	TEAE		TRAE	
Adverse event, *n* (%)	All grades	Grades 3/4/5	All grades	Grades 3/4/5
Nausea	23 (79)	0	21 (72)	0
Creatinine increased	16 (55)	0	4 (14)	0
Anemia	15 (52)	3 (10)	7 (24)	1 (3)
Hyponatremia	15 (52)	5 (17)	9 (31)	3 (10)
Platelet count decreased	14 (48)	0	10 (34)	0
Fatigue	12 (41)	2 (7)	10 (34)	1 (3)
Vomiting	11 (38)	0	10 (34)	0
White blood cell decreased	11 (38)	2 (7)	8 (28)	1 (3)
Hyperkalemia	10 (34)	0	1 (3)	0
Anorexia	9 (31)	0	7 (24)	0
Dyspnea	9 (31)	0	0	0
Aspartate aminotransferase increased	7 (24)	2 (7)	1 (3)	0
Diarrhea	6 (21)	1 (3)	3 (10)	0
Dizziness	6 (21)	0	1 (3)	0
Hyperuricemia	6 (21)	0	1 (3)	0
Neutrophil count decreased	6 (21)	2 (7)	5 (17)	2 (7)
Alanine aminotransferase increased	5 (17)	2 (7)	1 (3)	0
Cough	5 (17)	0	0	0
Hypophosphatemia	5 (17)	2 (7)	0	0
Hypothyroidism	5 (17)	0	0	0
Rash maculo‐papular	5 (17)	0	0	0
Urinary frequency	5 (17)	0	0	0
Blurred vision	4 (14)	0	2 (7)	0
Constipation	4 (14)	0	1 (3)	0
General disorders and administration site conditions	4 (14)	1 (3)	1 (3)	0
Investigations—Other	4 (14)	1 (3)	0	0
Lipase increased	4 (14)	2 (7)	1 (3)	0
Thromboembolic event	4 (14)	4 (14)	2 (7)	2 (7)
Weight loss	4 (14)	0	2 (7)	0
Adrenal insufficiency	3 (10)	2 (7)	1 (3)	1 (3)
Cataract	3 (10)	0	2 (7)	0
Confusion	3 (10)	0	2 (7)	0
Fall	3 (10)	0	0	0
Fever	3 (10)	0	1 (3)	0
Gastrointestinal disorders—Other, specify	3 (10)	0	0	0
Hypomagnesemia	3 (10)	0	0	0
Lymphocyte count decreased	3 (10)	0	0	0
Nasal congestion	3 (10)	0	0	0
Serum amylase increased	3 (10)	0	0	0
Acute kidney injury	2 (7)	1 (3)	1 (3)	1 (3)
Alkaline phosphatase increased	2 (7)	0	0	0
Arthralgia	2 (7)	1 (3)	0	0
Back pain	2 (7)	0	0	0
Blood bilirubin increased	2 (7)	1 (3)	0	0
Chest wall pain	2 (7)	1 (3)	0	0
Dysgeusia	2 (7)	0	2 (7)	0
Flashing lights	2 (7)	0	1 (3)	0
Gait disturbance	2 (7)	0	0	0
Headache	2 (7)	0	1 (3)	0
Hematuria	2 (7)	0	0	0
Hyperglycemia	2 (7)	1 (3)	0	0
Hyperhidrosis	2 (7)	0	1 (3)	0
Hypocalcemia	2 (7)	0	0	0
Infections and infestations	2 (7)	1 (3)	0	0
Lung infection	2 (7)	0	0	0
Myalgia	2 (7)	0	0	0
Pain	2 (7)	0	0	0
Periorbital edema	2 (7)	0	0	0
Peripheral sensory neuropathy	2 (7)	0	0	0
Proteinuria	2 (7)	0	0	0
Pruritus	2 (7)	0	0	0
Respiratory, thoracic, and mediastinal disorders—Other, specify	2 (7)	0	0	0
Abdominal pain	1 (3)	0	0	0
Activated partial thromboplastin time prolonged	1 (3)	0	0	0
Alopecia	1 (3)	0	1 (3)	0
Arthritis	1 (3)	0	0	0
Ataxia	1 (3)	0	0	0
Atrial fibrillation	1 (3)	0	0	0
Atrial flutter	1 (3)	1 (3)	0	0
Bruising	1 (3)	0	0	0
CPK increased	1 (3)	0	0	0
Chest pain—cardiac	1 (3)	0	0	0
Chills	1 (3)	0	1 (3)	0
Dehydration	1 (3)	0	1 (3)	0
Delirium	1 (3)	0	1 (3)	0
Dysphagia	1 (3)	0	0	0
Edema face	1 (3)	0	0	0
Edema limbs	1 (3)	0	0	0
Endocrine disorders—Other, specify	1 (3)	1 (3)	0	0
Flank pain	1 (3)	0	0	0
Generalized muscle weakness	1 (3)	0	0	0
Hypercalcemia	1 (3)	0	0	0
Hyperthyroidism	1 (3)	0	0	0
Hypoalbuminemia	1 (3)	0	0	0
Hypoglycemia	1 (3)	0	0	0
Hypokalemia	1 (3)	0	0	0
Hypotension	1 (3)	0	1 (3)	0
INR increased	1 (3)	0	0	0
Insomnia	1 (3)	0	0	0
Joint effusion	1 (3)	1 (3)	0	0
Metabolism and nutrition disorders—Other, specify	1 (3)	0	0	0
Mucositis oral	1 (3)	0	0	0
Musculoskeletal and connective tissue disorder—Other, specify	1 (3)	0	0	0
Nervous system disorders—Other, specify	1 (3)	0	0	0
Pain in extremity	1 (3)	0	0	0
Pneumonitis	1 (3)	1 (3)	0	0
Postnasal drip	1 (3)	0	0	0
Productive cough	1 (3)	0	0	0
Rash acneiform	1 (3)	0	0	0
Renal and urinary disorders—Other, specify	1 (3)	0	0	0
Sepsis	1 (3)	0	0	0
Skin and subcutaneous tissue disorders—Other, specify	1 (3)	0	0	0
Somnolence	1 (3)	0	0	0
Sore throat	1 (3)	0	0	0
Stroke	1 (3)	0	1 (3)	0
Surgical and medical procedures—Other, specify	1 (3)	1 (3)	0	0
Syncope	1 (3)	1 (3)	0	0
Urinary retention	1 (3)	0	0	0
Urinary tract infection	1 (3)	1 (3)	0	0
Urinary tract pain	1 (3)	0	0	0
Vertigo	1 (3)	0	0	0

### 3.3 Antitumor Activity

At a median follow‐up of 12.4 months, 27 patients were evaluable for response (Table 4). Five patients (19%) achieved a partial response (PR), 17 (63%) experienced stable disease (SD), and five (19%) had progressive disease (PD). CBR was 65% (*n* = 15).

**TABLE 4:** Summary of best overall response.

Measure	All patients	Treatment arm	
		Selinexor + nivolumab + ipilimumab	Selinexor + nivolumab
Response, *n* (%)			
Evaluable patients	27	10	17^a^
CR	0	0	0
PR	5 (19)	2 (20)	3 (18)
Confirmed	4 (15)	1 (10)	3 (18)
Unconfirmed	1 (4)	1 (10)	0
SD	17 (63)	6 (60)	11 (65)
CBR (CR + PR + SD ≥ 6 months)	15 (56)	5 (50)	10 (59)
DCR (CR + PR + SD)	22 (81)	8 (80)	14 (82)
PD	5 (19)	2 (20)	3 (18)
TTF in weeks			
Evaluable patients	29	10	19
median (range)	47.7 (5.7–107.8)	35.8 (5.7–53.8)	57.8 (6.5–107.8)
CBR patients			
Number of patients	15	5	10
Median (range)	61.4 (47.4–107.8)	52.7 (47.4–53.8)	74.8 (56.9–107.8)
Number of cycles			
Evaluable patients	29	10	19
Median (range)	7 (1–20)	7 (3–14)	6 (1–20)
CBR patients			
Number of patients	15	5	10
Median (range)	13 (1–20)	8 (3–14)	15.5 (1–20)

Abbreviations: CBR, clinical benefit rate; DCR, disease control rate; TFT, time‐to‐treatment failure.

^a^ Two patients in the nivolumab alone arm had unknown response.

**FIGURE 3:**
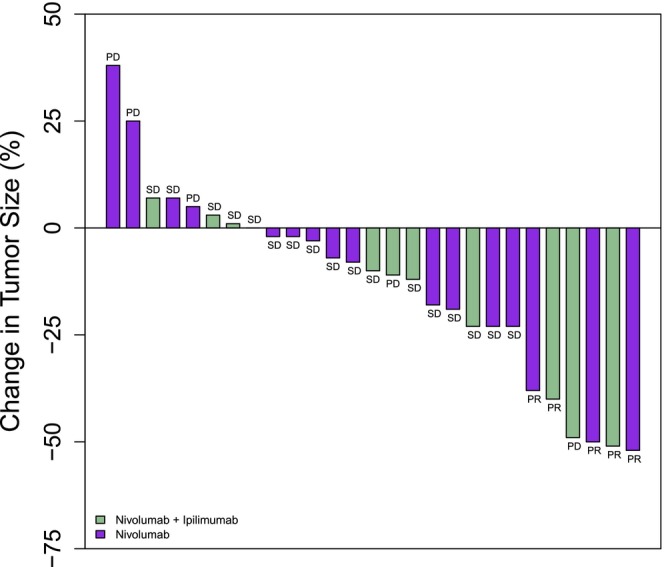
Waterfall plot showing the best percent change in tumor diameter (per RECIST v1.1) for patients who received selinexor in addition to nivolumab (purple) or nivolumab/ipilimumab (green). In 27 patients who were evaluable for response, five (19%) patients achieved a partial response (PR), 17 (63%) experienced stable disease (SD), and five (19%) had progressive disease (PD). * All patients received selinexor per study protocol.

**FIGURE 4:**
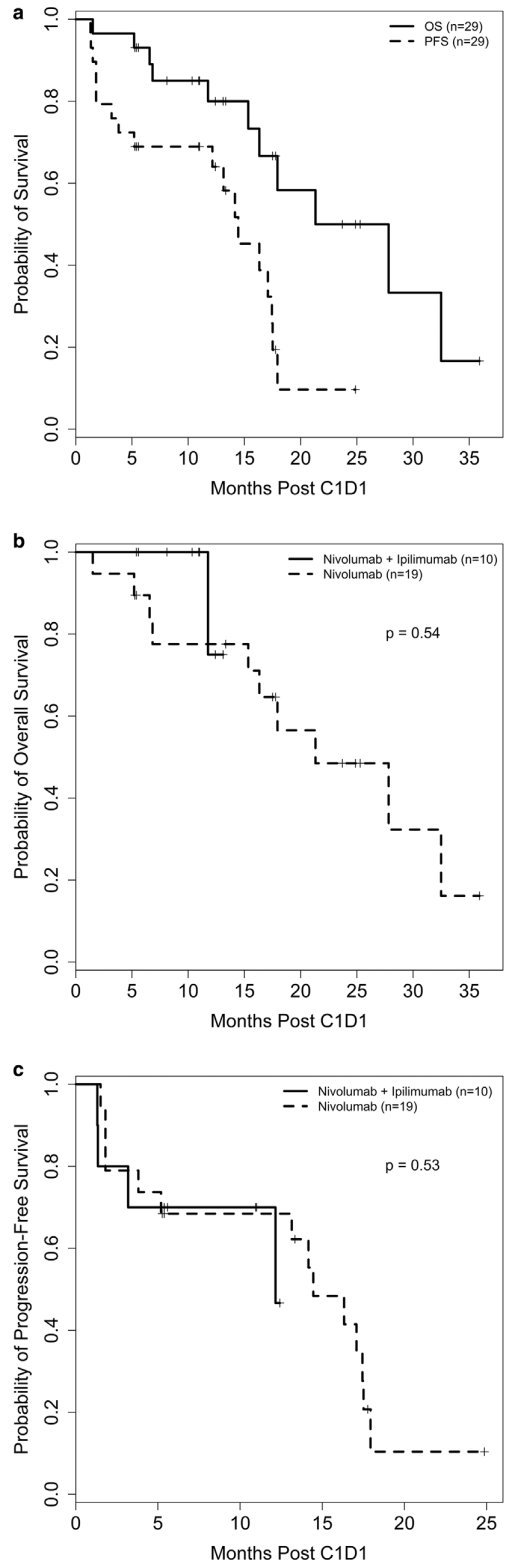
Overall survival and progression‐free survival in included patients who received treatment with selinexor in combination with nivolumab or nivolumab plus ipilimumab. (a) Overall and progression‐free survival for All patients. Median (95% CI): OS, 27.8 (15.3, 32.5) months; PFS, 14.5 (5.2, 17.4) months. (b) Overall survival—by treatment arm. Median (95% CI): Nivo+Ipi, 27.8 (11.8, not estimated [NE]) months; Nivo, 21.3 (6.6, NE) months. (c) Progression‐free survival—by treatment arm. Median (95% CI): Nivo+Ipi, 13.1 (1.8, 17.5) months; Nivo, 16.3 (1.8, 17.4) months.

Among the patients with ccRCC who received SEL + NIVO/IPI as a first‐line (1 L) therapy (*N* = 4), all (100%) had SD as the best overall response. Among the patients with ccRCC who received SEL + NIVO as 1 L (*N* = 2), one patient achieved a confirmed PR and the other experienced SD. The median number of cycles was 7 (range 1–20). Among evaluable patients who had a prior anti‐PD‐1/L1 (*N* = 8), one achieved PR (unconfirmed), four had SD, and three had PD. Best overall tumor response among 27 evaluable patients is depicted in Figure 3. The median PFS for all patients was 14.5 months (95% CI 5.2, 17.4) (Figure 4), and the median OS for all patients was 27.8 months (95% CI 15.3, 32.5) (Figure 4).

## 4 Discussion

This study reports on the combination of selinexor with ICB. This phase 1b trial demonstrated the combination of selinexor and NIVO or NIVO/IPI was tolerable. However, most patients treated with the combination regimen had at least one TEAE. Consistent with other studies of selinexor, the most commonly reported adverse events were hematologic toxicities, gastrointestinal adverse events, and constitutional symptoms [27]. Overall, in this study, most toxicities were low grade and could be managed with the use of supportive measures. Serious adverse events were experienced in 28% of patients. In our study, there was no increase in Grade 3 or 4 bone marrow toxicity as compared to other trials investigating selinexor combined with chemotherapy [27, 28, 29, 30] possibly due to the nonoverlapping toxicity between selinexor and ICB agents.

There were limited radiographic responses to the combination in patients with prior ICB which is compatible with recent data suggesting that rechallenge with immunotherapy in this scenario is unlikely to be beneficial [31]. Furthermore, initial studies of selinexor monotherapy showed modest response rates. For example, the first in human trial suggested an ORR of 4%, which did not include any patients with RCC [20]. However, PFS was 14.5 months in this population where 41% received trial therapy as their third line or beyond. PFS in this scenario is typically estimated in 3–5 months [32]. Therefore, the rate of clinical benefit from the combination remains noteworthy. Perhaps this highlights a potential role for selinexor in maintenance setting or earlier in the disease course. The small number of patients in this study warrants interpretation of efficacy results with caution; and it remains unclear if a true additive or synergistic benefit exists with ICB in RCC. Future work should help to address such questions and would entail the conduct of larger clinical trials designed to validate findings from this study.

## 5 Conclusion

The XPO1 inhibitor, selinexor, in combination with ICB had a favorable safety profile with no added toxicity concerns. However, responses were limited to patients without prior ICB exposure. Further evaluation maybe warranted, especially in an ICB‐naïve populations.

## Author Contributions

**Omar Alhalabi:** data curation (lead), writing – original draft (lead), writing – review and editing (lead). **Mohamed A. Gouda:** data curation (equal), writing – original draft (equal), writing – review and editing (equal). **Denái R. Milton:** formal analysis (lead), methodology (lead), writing – review and editing (equal). **Hassan Ahmed Momin:** data curation (equal), writing – review and editing (equal). **Bulent Yilmaz:** data curation (equal), writing – review and editing (equal). **Bettzy Stephen:** data curation (equal), project administration (lead), writing – review and editing (equal). **Chinenye Lynette Ejezie:** data curation (equal). **Justin Tyler Moyers:** investigation (equal), writing – review and editing (equal). **Serdar A. Gurses:** investigation (equal), writing – review and editing (equal). **Jeffrey How:** investigation (equal), writing – review and editing (equal). **Siqing Fu:** investigation (equal), writing – review and editing (equal). **Jordi Rodon Ahnert:** investigation (equal), writing – review and editing (equal). **David S. Hong:** investigation (equal), writing – review and editing (equal). **Sarina A. Piha‐Paul:** investigation (equal), writing – review and editing (equal). **Vivek Subbiah:** investigation (equal), writing – review and editing (equal). **Ecaterina Elena Dumbrava:** investigation (equal), writing – review and editing (equal). **Daniel D. Karp:** investigation (equal), writing – review and editing (equal). **Filip Janku:** investigation (equal), writing – review and editing (equal). **Funda Meric‐Bernstam:** investigation (equal), writing – review and editing (equal). **Nizar M. Tannir:** investigation (lead), writing – original draft (lead), writing – review and editing (lead). **Aung Naing:** conceptualization (lead), data curation (lead), investigation (lead), writing – original draft (lead), writing – review and editing (lead).

## Ethics Statement

Ethical approval was obtained from the institutional review board at The University of Texas MD Anderson Cancer Center.

## Consent

All patients signed informed consent prior to starting trial procedures.

## Conflicts of Interest

Justin Tyler Moyers reports being on the advisory board for Replimmune. Siqing Fu reports clinical Trial Research Support/Grant Funding through the institution from the following sources: NIH/NCI P30CA016672—Core Grant (CCSG Shared Resources); Abbisko; Antengene; BeiGene; BeyongSpring Pharmaceuticals Inc.; BioAtla LLC.; Boehringer Ingelheim; CUE Biopharma Inc.; DEKA Biosciences; Eli Lilly & Co.; Exelixis; Greenfire Bio Inc.; Hookipa Biotech; IMV Inc.; Innovent Biologics, Co. Ltd.; Jazz Pharmaceuticlals; K‐Group Beta; Lantern Pharma Inc.; Lyvgen Biopharm, Co. Ltd.; MacroGenics; MediLink Therapeutics, Co. Ltd.; Millennium Pharmaceuticals Inc.; Nerviano Medical Sciences; NeuPharma Inc.; NextCure Inc.; Ningbo NewBay Technology Development Co. Ltd.; Novartis; NovoCure; Nykode Therapeutics AS.; Parexel International LLC; PharmaMar USA Inc.; Pionyr Immunotherapeutics Inc.; PureTech Health LLC; Qurgen Inc.; Shanghai Huaota Biopharmaceutical Co. Ltd.; Sellas Life Sciences Group; Soricimed Biopharma Inc.; SQZ Biotechnologies; Sumitomo Dainippon; Taiho Oncology and NCCN; Treadwell Therapeutics; Turnstone Biologics; Tyligand Bioscience Ltd.; Virogin Biotech Ltd. Sarina Anne Piha‐Paul reports clinical trial research support/grant Funding through the institution from AbbVie Inc.; ABM Therapeutics Inc.; Acepodia Inc.; Alkermes; Aminex Therapeutics; BioMarin Pharmaceutical Inc.; Boehringer Ingelheim; Bristol Myers Squib; Cerulean Pharma Inc.; Chugai Pharmaceutical Co. Ltd.; Curis Inc.; Cyclacel Pharmaceuticals; Daiichi Sankyo; Eli Lilly; ENB Therapeutics; Epigenetix Inc.; Five Prime Therapeutics; F‐Star Beta Limited; F‐Star Therapeutics; Gene Quantum; Genmab A/S; Gilead Sciences Inc.; GlaxoSmithKline; Helix BioPharma Corp.; Hengrui Pharmaceuticals, Co. Ltd.; HiberCell Inc.; Immunomedics Inc.; Incyte Corp.; Jacobio Pharmaceuticals Co. Ltd.; Jiangsu Simcere Pharmaceutical Co. Ltd.; Loxo Oncology Inc.; Lytix Biopharma AS; Medimmune LLC.; Medivation Inc.; Merck Sharp and Dohme Corp.; Nectin Therapeutics Ltd.; Novartis Pharmaceuticals; Nurix; Pieris Pharmaceuticals Inc.; Pfizer; Phanes Therapeutics; Principia Biopharma Inc.; Puma Biotechnology Inc.; Purinomia Biotech Inc.; Rapt Therapeutics Inc.; Replimune; Roche/Blueprint; Seattle Genetics; Silverback Therapeutics; Shasqi Inc.; Synlogic Therapeutics; Taiho Oncology; Tesaro Inc.; Theradex Oncology; Toragen Therapeutics Inc.; TransThera Bio; Xencor Inc.; ZielBio Inc.; NCI/NIH; P30CA016672—Core Grant (CCSG Shared Resources); and working as a consultant for CRC Oncology. Jordi Rodon reports nonfinancial support and reasonable reimbursement for travel from European Society for Medical Oncology and Loxo Oncology; receiving consulting and travel fees from Ellipses Pharma, Molecular Partners, IONCTURA, Sardona, Mekanistic, Amgen, Merus, MonteRosa, Aadi, and Bridgebio (including serving on the scientific advisory board); Consulting fees from Vall d'Hebron Institute of Oncology/Ministero De Empleo Y Seguridad Social, Chinese University of Hong Kong, Boxer Capital LLC, Tang Advisors LLC and Guidepoint, receiving research funding from Blueprint Medicines, Merck Sharp & Dohme, Hummingbird, AstraZenneca, Yingli, Vall d'Hebron Institute of Oncology/Cancer Core Europe; and serving as investigator in clinical trials with Cancer Core Europe, Symphogen, BioAlta, Pfizer, Kelun‐Biotech, GlaxoSmithKline, Taiho, Roche Pharmaceuticals, Hummingbird, Yingli, Bicycle Therapeutics, Merus, AadiBioscience, ForeBio, Loxo Oncology, Hutchinson MediPharma, Ideaya, Amgen, Tango Therapeutics, Mirati, Linnaeus Therapeutics, MonteRosa, Kinnate, Yingli, Debio, BioTheryX, Storm Therapeutics, Beigene, MapKure, Relay, Novartis, FusionPharma, C4 Therapeutics, Scorpion Therapeutics, Incyte, Fog Pharmaceuticals, Tyra, Nuvectis Pharma. Funda Meric‐Bernstam reports consulting for AbbVie, Aduro BioTech Inc., Alkermes, AstraZeneca, Daiichi Sankyo Co. Ltd., Calibr (a division of Scripps Research), DebioPharm, Ecor1 Capital, eFFECTOR Therapeutics, F. Hoffman‐La Roche Ltd., GT Apeiron, Genentech Inc., Harbinger Health, IBM Watson, Incyte, Infinity Pharmaceuticals, Jackson Laboratory, Kolon Life Science, LegoChem Bio, Lengo Therapeutics, Menarini Group, OrigiMed, PACT Pharma, Parexel International, Pfizer Inc., Protai Bio Ltd., Samsung Bioepis, Seattle Genetics Inc., Tallac Therapeutics, Tyra Biosciences, Xencor, Zymeworks; advisory Committee membership for Black Diamond, Biovica, Eisai, FogPharma, Immunomedics, Inflection Biosciences, Karyopharm Therapeutics, Loxo Oncology, Mersana Therapeutics, OnCusp Therapeutics, Puma Biotechnology Inc., Seattle Genetics, Sanofi, Silverback Therapeutics, Spectrum Pharmaceuticals, Theratechnologies, Zentalis; Sponsored Research (to the institution) from Aileron Therapeutics Inc. AstraZeneca, Bayer Healthcare Pharmaceutical, Calithera Biosciences Inc., Curis Inc., CytomX Therapeutics Inc., Daiichi Sankyo Co. Ltd., Debiopharm International, eFFECTOR Therapeutics, Genentech Inc., Guardant Health Inc., Klus Pharma, Takeda Pharmaceutical, Novartis, Puma Biotechnology Inc., Taiho Pharmaceutical Co.; Honoraria from Dava Oncology; and Other (Travel Related) from European Organization for Research and Treatment of Cancer (EORTC), European Society for Medical Oncology (ESMO), Cholangiocarcinoma Foundation, Dava Oncology. Nizar M. Tannir reports honorarium from AstraZeneca (Consulting/advisory), Bristol‐Myers‐Squibb (Consulting/advisory meeting), Eisai Medical Research (Consulting/advisory), Exelixis (Consulting/advisory), lntellisphere (Consulting/advisory), Merck Sharp & Dohme (Consulting/advisory), Nektar Therapeutics (Consulting/advisory), and Oncorena (Consulting/advisory); and clinical grants from Bristol‐Myers‐Squibb (Institutional), Calithera Biosciences (Institutional), Exelixis (Institutional), Nektar Therapeutics (Institutional), and Novartis (Institutional). Aung Naing reports research funding from NCI, EMD Serono, MedImmune, Healios Onc. Nutrition, Atterocor/Millendo, Amplimmune, ARMO BioSciences, Karyopharm Therapeutics, Incyte, Novartis, Regeneron, Merck, Bristol‐Myers Squibb, Pfizer, CytomX Therapeutics, Neon Therapeutics, Calithera Biosciences, TopAlliance Biosciences, Eli Lilly, Kymab, PsiOxus, Arcus Biosciences, NeoImmuneTech, Immune‐Onc Therapeutics, Surface Oncology, Monopteros Therapeutics, BioNTech SE, Seven & Eight Biopharma, and SOTIO Biotech AG; being on advisory board/receiving consulting fees from CTI, Deka Biosciences, Janssen Biotech, NGM Bio, PsiOxus Therapeutics, Immune‐Onc Therapeutics, STCube Pharmaceuticals, OncoSec KEYNOTE‐695, Genome & Company, CytomX Therapeutics, Nouscom, Merck Sharp & Dohme Corp, Servier, Lynx Health, AbbVie, PsiOxus; travel and accommodation expense from ARMO BioSciences, NeoImmuneTech, NGM Biopharmaceuticals; honoraria for speaking engagements from AKH Inc., The Lynx Group, Society for Immunotherapy of Cancer (SITC), Korean Society of Medical Oncology (KSMO), Scripps Cancer Care Symposium, ASCO Direct Oncology Highlights, European Society for Medical Oncology (ESMO), CME Outfitters. Remaining authors declared no conflicts of interest.

## Précis

Treatment with selinexor in combination with nivolumab monotherapy or nivolumab plus ipilimumab is well‐tolerated. In patients with advanced renal cell carcinoma, the combination showed modest clinical activity.

## Data Availability

Data can be available upon reasonable request from corresponding author.
